# The coproduction of a multilevel personal narrative intervention for people with aphasia in a community communication support group—A pilot study

**DOI:** 10.3389/fstro.2024.1393676

**Published:** 2024-07-17

**Authors:** Marina Charalambous, Rafaella Tereza Symeou, Elena Theodorou, Maria Kambanaros

**Affiliations:** Department of Rehabilitation Sciences, School of Health Sciences, Cyprus University of Technology, Limassol, Cyprus

**Keywords:** stroke, people with aphasia, personal narrative skills, aphasia communication group, coproduction

## Abstract

**Introduction:**

People with aphasia (PWA) face challenges in sharing personal stories due to communication difficulties. Discourse treatment in aphasia focusing on personal narrative macrostructure has yet to receive the attention this warrants of researchers and clinicians. Emerging person-centered approaches involve coproduction and codesign with PWA for meaningful discourse treatments. Few studies explore discourse treatment's impact on functional communication. This pilot study aims to explore whether the use of the coproduction approach in the development of a multilevel personal narrative intervention at the group level increased the production of macrostructure elements in trained and untrained narrative discourse contexts, improved aphasia severity and functional communication skills, and advanced quality of life of the participants with aphasia.

**Methods:**

An ABA design was followed featuring a pre-treatment baseline assessment phase, a treatment phase, and a post-treatment assessment phase immediately after treatment was completed. Three people with chronic stroke-induced aphasia, three communication partners, and a moderator took part in the study. All participants were members of a university-led community aphasia communication group. The research protocol consisted of eleven, two-hour, weekly sessions over an 11-week block. Nine treatment sessions were carried out following codesign and coproduction methods that focused on participants with aphasia producing words, sentences, and total communication strategies to express macrostructure elements in their personal stories. Assessment measures were collected at baseline and post-treatment to evaluate improvements in trained and untrained narrative abilities, aphasia severity, functional communication, and the impact of aphasia on quality of life.

**Results:**

Multilevel personal narrative therapy improved the narrative skills of the participants with aphasia at the macrostructural level of narrative discourse. Improvements were also observed in functional communication and quality of life post-treatment.

**Discussion:**

The involvement of participants with aphasia in the codesign and coproduction of the treatment content for the group intervention facilitated improvement in narrative skills, functional communication, and overall quality of life with aphasia. It is recommended that researchers and clinicians consider using content from the personal narratives of clients with aphasia to build discourse treatment and adopt codesign and coproduction approaches, when designing interventions for people with chronic aphasia, to improve communication outcomes in everyday life.

## Introduction

Personal story telling is the cornerstone of human communication. It is the vehicle that drives speakers to convey complex ideas, create connections, evoke emotions, build trust and empathy and gain enjoyment from interacting with other people. People with aphasia (PWA), an acquired communication impairment, caused by damage to the language networks in the brain, usually because of stroke (Berg et al., 2020), often have difficulties telling personal stories and this creates significant barriers to meaningful participation in family life (Killmer et al., 2022), community events (Kim et al., 2023), in maintaining social networks and friendship circles (Doedens and Meteyard, 2019; Manning et al., 2019; Azios et al., 2021), and for return to the workforce (Gilmore et al., 2022). For people with chronic aphasia, difficulties with real-life discourse can lead to social isolation (Hoover et al., 2020), chronic mood disorders including depression (Laures-Gore et al., 2020), reduced quality of life (Charalambous et al., 2022a) and poorer overall functional recovery (Ali et al., 2015).

Personal narratives are fundamental components of discourse. Linguistically, discourse extends beyond individual sentences and encompasses language usage in everyday contexts, reflecting its functional aspect (Stubbs, 1983). Narrative discourse can be analyzed at micro and macrostructural levels with the former involving analyses at the levels of phonology, lexical-semantics and syntax and the latter at the story level including conceptual representation and global meaning (gist) (Boyle, 2011). Enhancing discourse skills can improve functional communication, benefiting individuals in social and professional contexts (Dipper et al., 2020). Therefore, addressing the macrostructure and discourse organization through targeted interventions can enhance affected individuals' communicative abilities and overall quality of life.

Generally speaking, for PWA, difficulties with narration result from a breakdown at the microlinguistic structure of discourse (e.g., use of lexical-semantics and grammatical forms) rather than the macrostructural level however microstructural impairments impact the macrostructural components underlying narrative construction (Boyle, 2011). For example, Kambanaros (2019) describes participants with chronic anomic aphasia demonstrating significant lexical retrieval (microstructure) difficulties when narrating their personal “stroke story” that affected their ability to complete the narrative in a coherent and cohesive (macrostructure) manner for the listener to understand the gist of the story.

In the first systematic review of research on the important topic of treatment for discourse production in aphasia, Dipper et al. (2020) categorized the studies reviewed into discourse treatment studies targeting different levels of language including the word-level, sentence level, the macrostructure level and the multi-level (see Dipper et al., 2020, and references within). Strong evidence for treatment gains following discourse treatment was at the microstructural level mainly in the number of new words used in discourse/narration post-treatment and was the most common outcome measure across the studies.

Of interest to the present study was the finding that of the 25 studies reviewed by Dipper et al. (2020), only six studies reported gains in discourse macrostructure after treatment either explicitly targeting discourse macrostructure, that is, the overall story structure and information (Osiejuk, 1991; Carragher et al., 2015) or after multilevel therapies focusing on any combination of two of the levels, that is, single word level, and/or sentence level, and/or discourse level (Penn and Beecham, 1992; Dietz et al., 2018) or focusing on all three levels (single word, sentence, discourse) (Whitworth, 2010; Whitworth et al., 2015). Of these studies, only four reported face-to-face delivery of discourse treatment by a speech-language therapist (SLT) (Penn and Beecham, 1992; Whitworth, 2010; Carragher et al., 2015; Whitworth et al., 2015) and three studies based the treatment activities on some aspects of personal narrative content either selected by the participant with aphasia (Osiejuk, 1991; Dietz et al., 2018) or prompted by the clinician (e.g., “what did you do this morning”) (Penn and Beecham, 1992). In most cases (Osiejuk, 1991; Whitworth, 2010; Carragher et al., 2015; Whitworth et al., 2015), the goal of discourse treatment was to improve story grammar elements (i.e., setting, initiating event, direct consequence, etc.). Moreover, discourse treatment in the above-mentioned studies was primarily delivered individually (Osiejuk, 1991; Penn and Beecham, 1992; Whitworth, 2010; Whitworth et al., 2015; Dietz et al., 2018) or in dyads with a communication partner (Carragher et al., 2015) and no study had an outcome measure to formerly assess post-treatment improvement in functional communication in everyday life.

The present study focuses on building personal story-based interventions for PWA in the chronic stage of living with aphasia. Research so far on personal narrative work in aphasia is primarily geared toward the constructs of identity and identity renegotiation, and helping clients move forward with life after stroke and aphasia (Strong and Shadden, 2020). Yet the power and uniqueness of personal narratives, fundamental communicative activities, with the authorship of the story belonging to the person with aphasia (Strong and Shadden, 2020), renders them worthy of further exploration in the context of discourse treatment that is person-centered. The LUNA framework (Linguistic Underpinnings of Narratives in Aphasia) (Cruice et al., 2022) of narrative assessment and intervention is a first step in this direction building on principles of coproduction when developing discourse treatment based on the personal stories of PWA. Furthermore, using people's experiences of aphasia to codesign discourse treatment content that is meaningful and worthwhile to the individual with aphasia (Cruice et al., 2022) aims to boost communicative confidence (Howe et al., 2023) and motivate active participation in treatment blocks.

Given that personal narratives are central to living successfully with aphasia and are directly related to social engagement and quality of life (Corsten et al., 2014; Kambanaros, 2019; Strong and Shadden, 2020), it is possible that narrative skills could be further enhanced using evidence-based discourse treatment approaches (Dipper et al., 2021) within an aphasia communication group (ACG) setting that will offer an opportunity for PWA to practice communication with peers (Mason et al., 2020), further build social interactions, and broaden friendship networks (Lanyon et al., 2018). In fact, ACGs themselves, could be considered a form of communication therapy given the emphasis on conversation and discourse activities (e.g., storytelling), social and/or psychological support, education about stroke and aphasia, participation in group events and activities and community integration (Charalambous and Kambanaros, 2021).

Group members of ACGs are provided with opportunities to exercise total communication skills, that is, the use of gestures, singing, drawing, writing and/or a combination of all the above (Elman, 2007) to effectively communicate in a safe environment. Communication is also facilitated with the use of communication aids such as writing boards, tablets, communication books and aphasia friendly materials (Wallace S. J. et al., 2023). The main topics of group discussion in ACGs are usually centered around learning about/refreshing knowledge on stroke and aphasia, connecting the information to members' own experiences, asking questions on topics of concern/interest, discussing living with stroke and aphasia, and sharing stories about life before aphasia (Charalambous and Kambanaros, 2021).

Taking all the issues addressed in the introduction into consideration, the present study aims to explore whether the use of the coproduction approach in the development of a multilevel personal narrative intervention at the group level (1) increased the production of macrostructure elements in trained and untrained narrative discourse contexts, (2) improved aphasia severity and functional communication skills, and (3) advanced quality of life of the participants with aphasia.

## Materials and methods

### Design

**Figure 1:**
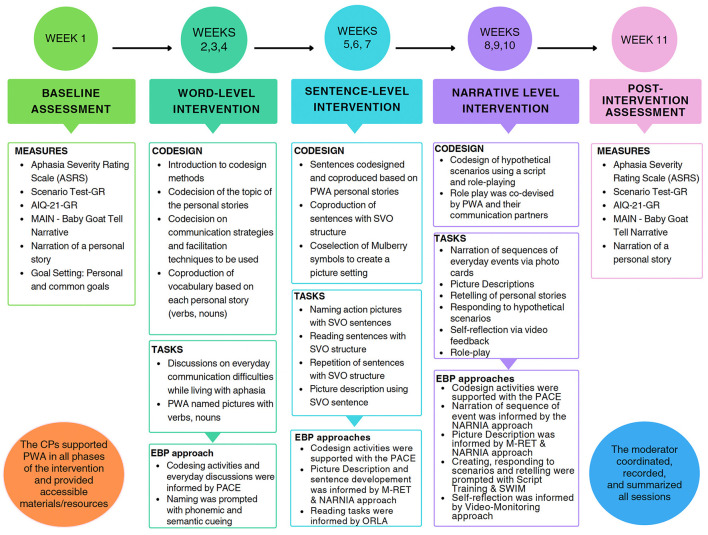
The study phases across the 11-week block. AIQ-21-GR, aphasia impact questionnaire greek version; MAIN, multilingual assessment instrument for narratives; PWA, people with aphasia; EBP, evidence based practice; M-Ret, modified response elaboration training; PACE, promoting aphasics' communicative effectiveness; ORLA, oral reading for language in aphasia; NARNIA, novel approach to real life communication; SWIM, someone who is not me; CPs, communication partners.

The study followed an ABA design with a pre-treatment baseline assessment phase, a treatment phase, and a post-treatment assessment phase conducted immediately after treatment was finished. Within this design, the researchers employed a multiple-case series approach to capture the individual performance of participants with aphasia within the intricate dynamics of an aphasia communication group (Crowe et al., 2011). This methodology proved fitting given the naturalistic and multifaceted context (real-world conditions) of the community aphasia communication support group. The study design is depicted graphically in Figure 1. The TIDieR (Template for Intervention Description and Replication) checklist (Hoffmann et al., 2014) was utilized for monitoring the necessary information to be included while describing the intervention (see Appendix 1).

### Setting

The research took place at the Cyprus University of Technology (CUT) Speech Therapy Rehabilitation Clinic in Limassol, Cyprus. The clinic hosts on a weekly basis, “The Aphasia Communication Team” (TACT), a community communication group that operates within the governance of the clinical internship of final-year speech-language therapy (SLT) students preparing to enter the profession. The TACT group involves people with chronic stroke-induced aphasia, and their communication partners, that is, the final-year SLT students (see Charalambous and Kambanaros, 2021 for a detailed description).

### Participants

Six participants were referred to the study from the TACT group between February 2022 and September 2022 with three participants later dropping out of the study. Two people with aphasia did not give consent to complete the study protocol and one dropped out due to illness. Bioethics approval was obtained from the Cyprus National Bioethics Committee (EEBK/EΠ/2017/37).

Three Cypriot Greek-speaking individuals with aphasia and three communication partners (final-year SLT students) took part in the study. A fourth final-year SLT student served as the ACG's moderator. Written informed consent was received from all participants before their involvement with the project.

### Participants with aphasia

Participants with aphasia met the following inclusion criteria: (1) native Greek speakers, (2) age ≥18 years, (3) had suffered a stroke at least 6 months before the study (chronic phase), and (4) presented with aphasia. Participants were excluded if they presented with (1) an additional diagnosis of dementia or any other degenerative disease, (2) profound hearing impairment and/or visual difficulties that would interfere with their performance in the study, and (3) a medical diagnosis of clinical depression or any other mental condition. Hearing, vision, and medical history were determined by observation, self-report, and/or reports from the carer during the case history interview.

The characteristics of the study participants with aphasia were as follows:

(1) Person with aphasia 1 (PWA1) was a 48-year-old, divorced woman with two children of Greek-Cypriot heritage, and a history of ischemic stroke related to recurrent atrial fibrillation. She presented with chronic moderate expressive aphasia. She lived with her daughter, had not returned to work after the stroke event, and was socially isolated.
(2) Person with aphasia 2 (PWA2) was a 68-year-old, retired public officer, and married with 3 children. He had five grandchildren and an active social life. He suffered an ischemic stroke several years ago, which left him with right hemiplegia and a mild to moderate anomic aphasia. He retired early from work after the stroke event.
(3) Person with aphasia 3 (PWA3) was a 60-year-old married man, with four children. He was a public employee and enjoyed hunting and dancing. He never returned to work or his recreational activities after his stroke. He presented with moderate anomic aphasia.

The demographics of the participants with aphasia are reported, using the DESCRIBE checklist (Wallace S. et al., 2023), in Table 1.

**Table 1:** Demographics of the participants with aphasia (DESCRIBE checklist).

**Characteristic**	**PWA1**	**PWA2**	**PWA3**
Age	48	68	60
Years of education	12	12	12
Biological sex	Female	Male	Male
Language of testing	Standard Modern Greek	Standard Modern Greek	Standard Modern Greek
Primary language	Standard Modern Greek^*^ and Cypriot-Greek Dialect^**^	Standard Modern Greek and Cypriot-Greek Dialect	Standard Modern Greek and Cypriot-Greek Dialect
Languages used in treatment	Standard Modern Greek and Cypriot-Greek Dialect	Standard Modern Greek and Cypriot-Greek Dialect	Standard Modern Greek and Cypriot-Greek Dialect
History of previous stroke	Ischemic	Ischemic	Ischemic
Lesion hemisphere	Left	Left	Left
Time since onset of aphasia	Five years	Seven years	Six years
Conditions arising from neurological event	Aphasia and mood disorders	Aphasia and hemiplegia	Aphasia and sensory impairment

PWA, person with aphasia.

^*^Standard Modern Greek = is the native language of Greeks living in Greece and is acquired (mainly used) at school in Cyprus, as it is the variety used in formal oral and written communication (Fotiou and Grohmann, 2022). Standard Modern Greek was used during formal testing.

^**^Cypriot Greek, a dialect of Standard Modern Greek, is the mother tongue of Greek Cypriots and is used in informal/ everyday interactions (Fotiou and Grohmann, 2022). Cypriot Greek was used during the group sessions and informal assessment procedures (personal stories).

### Communication partners and moderator

The demographics of the three communication partners (CPs) and the group's moderator are also reported following the DESCRIBE checklist (Wallace S. et al., 2023). The three CPs and the group's moderator were final-year SLT students in their final semester approaching entry-level to the profession. All the SLT students were identified as female and were 21 years old.

### Procedures

#### Baseline and post-treatment assessment measures

The following five measures were administered before the intervention as baseline measures and post-intervention as outcome measures:

(1) The *Aphasia Severity Rating Scale* (ASRS) from the Greek adaptation of the Boston Diagnostic Aphasia Examination Short Form (BDAE- SF) (Messinis et al., 2013) was used to rate the severity of the observed language and communication difficulties. Spontaneous speech samples were elicited during a 15-min semi-structured interview that comprised four topics: the stroke story, occupation, family, and hobbies (El Hachioui et al., 2014). Aphasia severity was assessed by the moderator of the group using the ASRS to allow a classification based on verbal output. Scores on the ASRS range from 0 to 5, with 0 revealing very severe non-fluent aphasia and 5 indicating very mild naming difficulties.
(2) *Spontaneous Language Samples*, that is, *personal narratives* were elicited from participants with aphasia by encouraging them to narrate three personal stories from childhood memory centered around their “House” in the village (birthplace). The three short stories functioned as baseline and post-treatment measures allowing three equal episodes to be compared to the untrained narrative task (picture-based story). Personal narratives were analyzed following the narrative macrostructure framework developed by Gagarina et al. (2019) that features the Setting, Initiating Event, Goal of the protagonist, Attempt, Outcome, and Internal State (Mental State Terms). Zero was given for wrong or no responses and one point was given for a correct response for each macrostructure element. Higher scores indicated complete macrostructure elements.
(3) A subtest of the *Multilingual Assessment Instrument for Narratives (MAIN)* (Gagarina et al., 2012), specifically, the “Baby goat” story was used to evaluate the telling of a story using picture support. The story includes a colorful six-picture sequence depicting a three-episode story involving different animals (goats, fox, bird) and a lake. The episodes contain carefully constructed goal-attempt-outcome sequences for the specific characters (Gagarina et al., 2019). Participants with aphasia were asked to narrate the story as illustrated in the pictures placed in front of them. The same stimulus story/materials and elicitation procedures were used with all three participants. A score from 0 to 17 points was given to each story produced. Zero was given for wrong or no responses and 1 point was given for a correct response for each macrostructure element (Initiating Event, Goal, Attempt, Outcome, Internal State Term). A higher score in the total number of macrostructure elements demonstrates the ability of the person to construct a story that can be interpreted as having better communication skills, coherence, and cohesion in conveying their message (Olness and Ulatowska, 2011).
(4) The standardized Greek-version of the *Scenario Test-GR* (Charalambous et al., 2022a) was used to measure functional communication. During the administration of the test, participants with aphasia were required to answer 18 questions related to six real–life scenarios (1) going shopping, (2) hailing a taxi (3) visiting a general practitioner (GP), (4) visiting a friend, (5) talking to the housekeeper and (6) ordering at a restaurant. Scores for each item range from 0 to 3, with 0 being a poor answer “Despite help not adequate or complete” and three being a correct answer with “No help needed.” A total score ranging from 0 to 54 is calculated, with higher scores indicating better functional communication performance.
  The standardized Greek version of the *Aphasia Impact Questionnaire-21 (AIQ-21-GR)* (Charalambous et al., 2022b) was administered to evaluate quality of life (QoL). This is a self-reported questionnaire that assesses the impact of aphasia on quality of life and includes 21 items related to three domains: Communication, Participation, and Emotional State/Well-being. Total scores range from 0 to 84, with higher scores indicating a higher impact of aphasia on QoL.

#### Personal and common treatment goals for participants with aphasia

Participants with aphasia identified various personal goals for treatment, each tailored to their individual needs and aspirations. Given treatment was provided in a group setting PWA also shared common goals. Both types of goals are reported in Table 2.

**Table 2:** The personal and common treatment goals for the participants with aphasia.

**Participant**	**Personal goal**	**Common goal**
*PWA1*	*Improving Communication Skills:*	*Enhancing Quality of Life:*
	PWA1 is focused on enhancing her ability to communicate effectively with her daughter despite facing language challenges. She aims to work on word finding, understanding others, and producing longer sentences (more than 2 words)	Through participation in the communication group, individuals with aphasia seek to regain a sense of normalcy, independence, and fulfillment in their daily interactions despite their communication difficulties
*PWA2*	*Building confidence:*	*Sharing experiences and coping strategies*:
	Aphasia has significantly affected the self-confidence and self-esteem of PWA2, particularly in family conversations. He believes that the group will provide a supportive environment where he and other members can practice communication without fear of judgment, thereby boosting their confidence levels. His specific aim is to engage more extensively in conversations with his family during mealtimes	Participants with aphasia aimed to share their experiences of living with chronic aphasia and exchange coping strategies, communication practical tips, and emotional support within the group
*PWA3*	*Social Connection:*	
	PWA3 highlights the impact of aphasia on his social connections due to communication difficulties. By participating in this group intervention, he seeks opportunities for social interaction and aims to foster connections with others who understand his communication challenges. Additionally, he aims to practice communication in everyday scenarios, such as ordering food	

PWA, person with aphasia.

### Treatment delivery and schedule

Group therapy was delivered face-to-face and consisted of nine sessions in total of 2-h duration, once weekly over 9 weeks (between weeks 2–10 inclusive) as described in the study phases in Figure 1.

The format of each treatment session was 90 min of treatment activities with two breaks of 15-min after every 45 min. The sessions were held in a group therapy room at the CUT Speech Therapy Rehabilitation clinic on a Wednesday afternoon. People with aphasia, their CPs, and the moderator were comfortably seated around a large table.

The first treatment session served as an introductory gathering for participants to get to know each other. Within this session, the ground rules for group work were negotiated collaboratively, ensuring that each member actively contributed their perspectives to shape the treatment. Participants were encouraged to converse and exchange information using any format (speech, gesture, drawing, writing, facial and body expressions etc.). The moderator noted all comments and preferences that were discussed within the group, where members were expected to be open about sharing their experiences, perspectives, and opinions (Cruice et al., 2022). They were also expected to listen respectfully to the experiences, perspectives, and opinions of others. The role of the moderator was to ensure equal weight of opinion across group members. The moderator was also tasked with simplifying language where necessary, keeping track of decisions made, and representing task outcomes visually using pictures or diagrams. She was also responsible for the organization of the social breaks in the session where group members had a break from treatment and shared refreshments.

To support communication and promote the engagement of PWA all written materials were developed in an aphasia-friendly format (Rose et al., 2012). Further strategies employed to enhance the engagement of PWA in the group sessions included: (a) asking concise and straightforward questions supported by an aphasia-friendly slide presentation; (b) utilizing whiteboards to record important words or phrases spoken by the participants or the CPs; (c) allowing additional time for responses; and (d) confirming participant responses promptly during the sessions (Dalemans et al., 2009; Wallace S. J. et al., 2023). Aphasia-friendly session notes were written for each session by the group moderator. The moderator circulated the session notes after each session by email to the communication partners and the authors of the paper and printed the notes in hard copy for participants with aphasia.

The BEFORE recommendations (Charalambous and Kambanaros, 2024) and the PAOLI (People with Aphasia and Other Layperson Involvement) framework were adopted to monitor the meaningful involvement of participants with aphasia in the study timeline (see Charalambous et al., 2023 for a full description of the PAOLI framework).

### Development of personal narrative treatment content following coproduction and codesign methods

**Figure 2:**
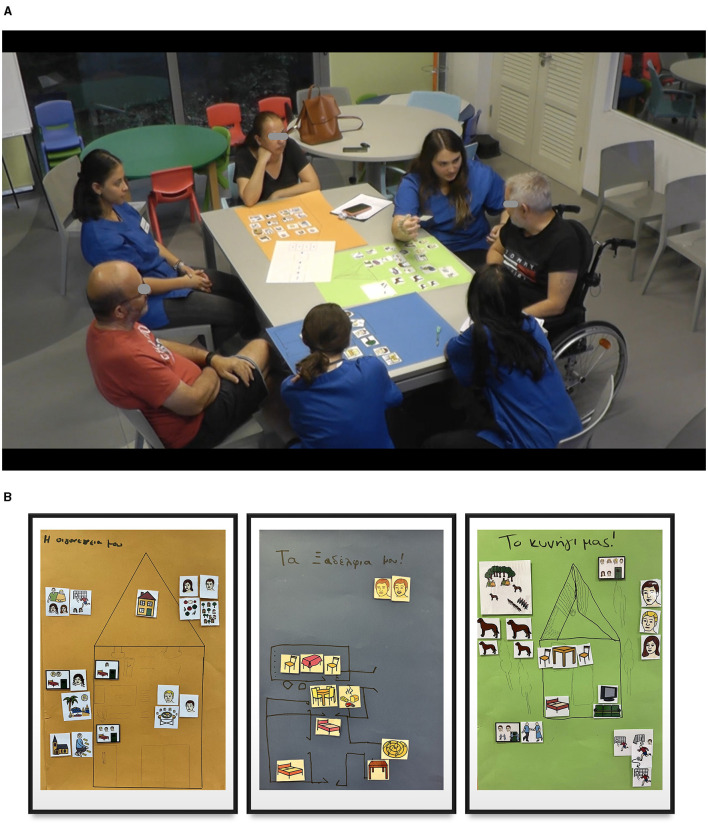
**(A)** The narration of personal stories while cocreating the “House” with the CPs. **(B)** The mockup of the three houses cocreated by PWA1, PWA2, PW3 (presented here in numerical sequence).

To develop the personal narrative treatment protocol, participants with aphasia in close collaboration with their CPs set out to create a 2D cardboard house and used this as a prop to narrate personal stories in relation to the verbal prompt: “Tell me a story about your house in the village.” This process is visually displayed in Figure 2A (photo) and the mockup of the three houses in Figure 2B.

The content of the intervention was based on the vocabulary and personal context of each participant when narrating stories of events around their “House.” Each session began with a review of the previous before introducing the topics and activities for the new session. This included refining key concepts/vocabulary as elicited from each person's personal story. For example, stories built from the “House” were given topic titles such as “My family,” “Going hunting,” and “Sunday lunch with my cousins.” Also, PWA were prompted to communicate additional personal events and experiences related to their personal stories (e.g., going grocery shopping, cleaning of my house, going to the village church, etc.) using a combination of photos, infographics, written words, and spoken language. After each session, the key points were summarized in aphasia-accessible format, later used for the recap/review segment of the next session. The key points included the facilitation strategies, the therapeutic techniques and approaches used, and the core vocabulary of the intervention produced from the events shared by the group.

The aim of embracing the codesign approach was to create a group intervention that was responsive to the unique needs, preferences, and experiences of the participants with aphasia, by fostering meaningful engagement, communication, and empowerment.

The codesign approach involved the active involvement of the three participants with aphasia, along with their CPs and the group's moderator, in the design and codevelopment of the group's structure, activities, and objectives. During the first session, the aim was to understand preferences and needs by conducting non-structured interviews to determine personal and common goals regarding the group intervention. This included selecting the topics of their personal story, their preferred communication style (total communication), and accessibility requirements (aphasia-friendly materials). During the codesign sessions (weeks 2–10) participants with aphasia and their CPs came together to brainstorm ideas, share insights, and collaboratively design the structure and content of the intervention. At this point, the moderator encouraged open communication and active participation from all group members. Furthermore, a flexible and inclusive design was followed to accommodate a diverse range of communication abilities and preferences among participants with aphasia. This involved incorporating alternative communication methods, such as visual prompts, communication books, and total communication strategies. Based on the input gathered from the participants with aphasia, the activities and topics designed for the intervention aimed to be relevant, engaging, and meaningful to participants with aphasia. This allowed for a variety of conversation formats, including storytelling, role-playing, group discussions, and creative expression activities. This approach empowered the participants with aphasia to take ownership of the conversation group by engaging in decision-making processes, facilitating leadership roles, and encouraging active participation in group facilitation and planning. Furthermore, a mechanism for ongoing evaluation and feedback to assess the effectiveness of the group intervention and identify areas for improvement was implemented by using an aphasia-friendly PowerPoint presentation at the beginning of each session to recap on what was completed until then and what needed to be changed. This encouraged open dialogue among participants with aphasia and CPs to continuously refine the group's design and activities based on evolving needs and preferences.

Several coproduction methods based on the LUNA protocol (Cruice et al., 2022) were followed in the treatment delivery. This included (1) open discussions about daily life and living with aphasia, e.g., challenges with ordering food or paying a bill, (2) brainstorming of thoughts and opinions on personal topics/events e.g., use of social media and visiting a friend, (3) direct questions and answers about current political and community happenings, (4) creative thinking exercises e.g., telephone pictionary, and (5) hypothetical scenarios about solving everyday communication problems from the perspective of another group member via the SWIM technique e.g., “How do you think Mary would respond to the email she received from her doctor?”.

### Evidence-based techniques and approaches used during personal narrative group intervention

Other evidence-based techniques and approaches were also used to facilitate expressive language and communication using the personal narrative content developed for each participant with aphasia during the nine-week intervention block. These methods, and those described above, commonly used in aphasia discourse treatment (Dipper et al., 2020) are reported in Table 3.

**Table 3:** A description of the techniques and approaches informing the intervention phase.

**Evidence-based techniques and approaches**	**Description**
Modified Response Elaboration Training (M-RET) (Wambaugh et al., 2013)	The CPs added linguistic elements to the utterances produced by participants with aphasia, to extend their sentences and provide a correct production model
Promoting Aphasics' Communicative Effectiveness (PACE) (Vitti and Hillis, 2021)	Participants with aphasia were encouraged by their CP to use gestures, drawing, and writing to produce a word, when expressive language was compromised
Oral Reading for Language in Aphasia (ORLA) (Cherney, 2010a,b)	Participants with aphasia read out loud sentences prepared by the CPs. These sentences were built by CP based on what PWA said in their personal stories
Novel Approach to Real Life Communication (NARNIA) (Whitworth et al., 2015)	The components of the sentences (subject, verb, object) that were extracted from the personal stories, were presented by the CP to PWA using the NARNIA diagram (WH Questions + pictures). In addition, discourse organization mind-maps and self-monitoring components have been utilized
Retelling (Cahana-Amitay and Jenkins, 2018)	Participants with aphasia were asked to repeat structured events that were narrated by the other group members. The CP's facilitated their responses with verbal prompting, phonological and semantic cueing
Script Training (ST) (Cherney et al., 2008)	Participants with aphasia produced spontaneous narratives that were related to hypothetical scenarios prepared by the CPs around activities of daily living (e.g., going to the supermarket etc.). The CP's facilitated their responses with visual prompting, and phonological and semantic cueing
“Someone Who Is Not Me” (SWIM) (Wilson et al., 2015)	Participants with aphasia were asked to respond to a hypothetical scenario from the perspective of another group member
Video monitoring (Roelofs, 2020)	Participants with aphasia were videoed during each session by the group moderator. They were given opportunities to watch their own videos, evaluate and reflect on their performance and discuss with the CPs and team members

### Intervention levels and therapeutic inputs

Personal narrative treatment was multilevel encompassing the word, sentence, and discourse levels over time as reported in Table 10 in Appendix 2. There were nine intervention sessions of which six sessions focused on the microstructure level, with target vocabulary content codesigned and coproduced with PWA and three sessions focused on treatment at the macrostructure level of narratives.

#### Single word level

During the first three treatment sessions (weeks 2–4 inclusive in Figure 1), PWA named pictures (Mulberry symbols) of verbs and nouns that emerged from the narration of their personal “House” story. For example, for PWA1 nouns included common objects (e.g., bed, table, chair, plate, cutlery, vegetables) and basic verbs (e.g., eat, play, read, sleep, study, work). For PWA2, nouns included common objects (e.g., bed, table, chairs, meat, cheese, bread) and verbs (e.g., eat, sleep, pray). For PWA3, nouns included common objects (e.g., bed, table, chairs, tv, couch, dogs, birds, football) and different verbs (e.g., hunt, dance, eat, play, visit). Total communication strategies, and the PACE technique were used to facilitate naming. Also, the CPs used several therapeutic inputs to encourage vocabulary development (e.g., prompting, modeling), and use of cueing (semantic and phonological) to aid word retrieval.

#### Sentence level

The focus of the three therapy sessions (weeks 5–7 inclusive in Figure 1) at the sentence level was to encourage participants with aphasia to produce SVO sentences. Participants were prompted to use sentences by describing pictures and reading out loud written sentences created by the CP based on the content from their personal stories and events e.g. “*My brother plays football*,” “*The dogs are eating meat*,” and “*My mother cooked roast*.” Therapeutic inputs used by the CPs included modeling the sentence for the participant with aphasia to repeat, prompting, and feedback.

#### Narrative discourse level

The final three treatment sessions (weeks 8–10 inclusive in Figure 1) targeted macrostructure elements. Participants with aphasia completed discourse activities using SVO structures when describing pictures, and photos showing a sequence of events. They engaged in hypothetical conversations using a script, practiced role-playing, and rehearsed retelling their personal stories. They also participated in informal discussions with their CPs and created individual everyday scripts based on their previous experiences related to their personal stories and their everyday lives when they were young.

### Content validity and participant satisfaction

The first author (MC), in consultation with the CPs, developed a 26-item self-rating questionnaire to assess the importance, comprehensiveness, relevance, and appropriateness of the therapy content. The questionnaire was divided into seven questions on the degree of relevance of the content of the intervention, seven questions on the appropriateness of the content of the intervention, and 12 questions on the significance of the content of the intervention. The questions were created following the Consensus-based Standards for the selection of health Measurement Instruments (COSMIN) guidelines (Terwee et al., 2018). Participants with aphasia, with the help of their CPs, completed the questionnaire using a 5-point Likert scale (1 “strongly disagree” to 5 “strongly agree”) to report on content validity. See a screenshot of the questionnaire in Appendix 3 providing an example of the scale and the presentation of the questions used in the content validity study. To assess the content of the intervention, analyses of the median scores were calculated. Likert scale data is best represented by the median due to its ordinal nature and the median's robustness to outliers, providing a more interpretable measure of central tendency than the mean. It was expected a median of 4 which shows that participants found the content as “very relevant.” Overall, results were expected to confirm the appropriate content of the intervention.

### Data collection and analysis

Baseline and post-treatment assessment data were collected and analyzed across the three standardized tools (ASRS, Scenario Test-GR, and the AIQ-21-GR) and the two language samples (personal story and the telling of the “Baby Goats” story) as reported in the Procedure section for each participant with aphasia. The data collection process was co-supervised by the first author (MC), a senior SLT specializing in the rehabilitation of post-stroke chronic aphasia, and the third author (ET) an academic SLT, specializing in narrative assessment and intervention. All sessions were video recorded, transcribed, and documented by the group moderator.

Descriptive statistics including mean scores and standard deviations were employed to present the results of the aphasia severity (ASRS), functional communication (Scenario Test-GR), aphasia impact measurements (AIQ-21-GR), and the language samples (personal story and Baby Goats), pre and post-intervention. Additionally, personal narratives and the telling of the “Baby Goats” story were analyzed at baseline and post-treatment following the narrative macrostructure framework developed by Gagarina et al. (2019) that incorporates the Setting, Initiating Event, Goal of the protagonist, Attempt, Outcome, and Internal State (Mental State Terms). Complete episodes were considered those that included a goal, attempt, and outcome (Gagarina et al., 2019). For the three short personal stories, the episodes were identified and analyzed based on the core elements of each story. This means that even if a single core element of a new story was mentioned in a story, it was classified as a separate episode for analysis purposes. This allowed three equal episodes to be compared with the three episodes of an untrained narrative task featuring “Baby Goats.” Although a picture-based story represents a different discourse genre, its structure shares the same elements as personal stories. Therefore, the expectation is that overall gains in one discourse genre may be seen in another connected speech task, albeit a task of reduced complexity.

All language samples were videoed and transcribed verbatim by authors MC and RTS. To ensure the rigor of the data analysis, the samples were analyzed and coded by authors RTS and ET independently. Minimum disagreements were resolved by discussion. An example of the analysis at the macrostructure level of a personal narrative, based on the Gagarina et al. (2019) framework is reported in Table 4.

**Table 4:** Macrostructure analysis of a story episode from the personal narrative of PWA1 at baseline.

**Spontaneous language sample of a personal story (1st episode)**	
«Σπίτι. Η οικογένεια, γονείς μου, κάθε μέρα επιέναν περβόλι. Εγώ τρεις αρφούες μου, Χρηστάκης, Νικόλας, Χριστόφορος, τρεις. Εθκιαβασε, μέστο πρώτη λυκείου» (Cypriot Greek)	
“Home (*setting*). The family, my parents, (*protagonist*) every day they went to the orchard (*initiating event*). Me and my three brothers, Chris, Nickolas, and Christopher, three. Was reading, in 1st grade.” (English translation)	
**Macrostructure elements**	**(1/0)**
Setting	1
Protagonist	1
Initiating event	1
Goal	0
Attempt	0
Outcome	0
Internal state	0

## Results

This study ran over 11 weeks where in the first and last week baseline and post-treatment assessment measures were administered respectively. Between weeks 2–10 inclusive, a total of nine, 2-h weekly group treatment sessions were completed over the 9 weeks.

Descriptive statistics for the scores on the ASRS, the Scenario Test-GR, and the AIQ-21-GR for the three PWA are presented in Table 5.

**Table 5:** Standardized test sub-scores and total scores at pre- and post-treatment.

	**PWA1**		**PWA2**		**PWA3**		**Mean (sd)**	
	**Pre-Tx**	**Post-Tx**	**Pre-Tx**	**Post-Tx**	**Pre-Tx**	**Post-Tx**	**Pre-Tx**	**Post-Tx**
*ASRS*	3	3	3	3	3	3	3.00 (n/a)	3.00 (n/a)
**Scenario test-GR sub scores**								
Shop	6	8	6	8	6	8	n/a	n/a
Taxi	6	7	7	8	6	8	n/a	n/a
Doctor	6	7	8	9	6	8	n/a	n/a
Visit	6	6	7	8	5	7	n/a	n/a
Housekeeper	6	6	6	7	6	7	n/a	n/a
Restaurant	6	8	6	7	6	6	n/a	n/a
*Scenario test-GR total scores*	36	42	44	47	35	44	38.3 (4.93)	44.3 (2.52)
**AIQ-21-GR sub-scores**								
Communication	16	9	9	4	13	3	n/a	n/a
Participation	6	5	8	7	8	2	n/a	n/a
Emotional/well-being	18	6	14	5	16	5	n/a	n/a
*AIQ-21-GR total scores*	40	20	31	16	37	10	36.0 (4.58)	15.3 (5.03)

PWA, people with aphasia; ASRS, Aphasia Severity Scale; AIQ-21-GR, Aphasia Impact Questionnaire Greek version; Tx, treatment; n/a, not applicable.

### Macrostructure elements of the personal narratives

Macrostructure analysis of the personal stories revealed different outcomes for each participant. Specifically, for PWA1 there was no change in the number of Goal, Attempt, and Outcome elements post-treatment, therefore no completed episodes were recorded. This participant produced a new initiating event for all three episodes without elaborating further and with no production of Internal State elements, neither at baseline nor post-intervention. PWA2 did not produce any Complete Episodes either at baseline or post-intervention. However, there was an increase in the production of Initiating Event, Goal, and Outcome elements. Similarly, for PWA3, there were no Complete Episodes. However, the production of the Initiating Event increased at the post-intervention assessment. The results in the macrostructure elements of the personal stories of PWA pre- and post-intervention are reported in Table 6. All three PWA were able to set the scene and introduce their protagonists, but they did not provide enough input on expanding the protagonists' actions.

**Table 6:** Number and type of macrostructure elements for the personal stories at baseline and post-intervention for participants with aphasia.

	**PWA1**		**PWA2**		**PWA3**		**Mean (sd)**	
	**Pre-Tx**	**Post- Tx**	**Pre-Tx**	**Post- Tx**	**Pre-Tx**	**Post- Tx**	**Pre-Tx**	**Post-Tx**
***Elements: Episode 1***								
Setting	1	1	1	1	1	1	n/a	n/a
Protagonist	1	1	1	1	1	1	n/a	n/a
Initiating event	1	0	1	1	1	1	n/a	n/a
Goal	0	0	1	1	0	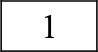	n/a	n/a
Attempt	0	0	0	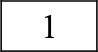	0	0	n/a	n/a
Outcome	0	0	0	0	0	0	n/a	n/a
Internal state	0	0	0	0	0	1	n/a	n/a
*Total macrostructure elements episode 1*	3	2	4	5	3	5	3.33 (0.57)	4 (1.73)
***Elements: Episode 2***								
Setting	1	1	1	1	1	1	n/a	n/a
Protagonist	1	1	1	1	1	1	n/a	n/a
Initiating event	1	1	0	0	1	1	n/a	n/a
Goal	0	0	0	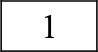	0	0	n/a	n/a
Attempt	0	0	0	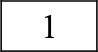	0	0	n/a	n/a
Outcome	0	0	0	0	0	0	n/a	n/a
Internal state	0	0	0	0	0	0	n/a	n/a
*Total macrostructure elements episode 2*	3	3	2	4	3	3	2.76 (0.57)	3.33 (0.57)
***Elements: Episode 3***								
Setting	1	1	1	1	1	1	n/a	n/a
Protagonist	1	1	1	1	1	1	n/a	n/a
Initiating event	1	0	0	1	0	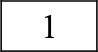	n/a	n/a
Goal	0	0	0	0	0	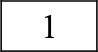	n/a	n/a
Attempt	0	0	0	0	0	0	n/a	n/a
Outcome	0	0	0	0	0	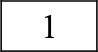	n/a	n/a
Internal state	0	1	0	0	0	0	n/a	n/a
*Total macrostructure elements episode 3*	3	3	2	3	2	5	2.33 (0.57)	3.67 (1.15)
***Total elements***	**9**	**8**	**8**	**12**	**8**	**13**	**8.33 (0.57)**	**11.0 (2.65)**

PWA, person with aphasia. Bold values indicate the total scores. The boxes indicate the changes in number of elements.

Overall, a numerical increase was seen in the production of macrostructure elements in all three episodes pre- (8.33, SD = 0.57) and post-intervention (11.0, SD = 2.65). The total elements produced pre- and post-intervention for PWA1 remained the same whereas, there was an increase in the number of 1 Attempt for PWA 2 during the narration of the first episode and 1 Attempt and 1 Goal during the narration of the second episode post-intervention. Also, PWA3 was the only participant who produced 1 Internal State element during the narration of his personal story post-intervention. The was no change in the production of Outcomes for any of the participants therefore no completed episodes were produced.

### Macrostructure elements from the telling of the “Baby Goats” story

Individual and total scores on the “Baby Goats” story of the MAIN at baseline and post-intervention assessment are shown in Table 7. According to the Gagarina et al. (2019) macrostructure analysis framework, the maximum score is 17 based on the number of elements available in the narrative. PWA1 scored 6/17 at baseline and 10/17 post-intervention, whereas PWA2, scored 9/17 pre- and post-intervention. In contrast, PWA3 received two points at baseline assessment and showed only a 1-point increase post-intervention. Overall, results suggest an improvement in macrostructure abilities for PWA1.

**Table 7:** Number and type of macrostructure elements for the “Baby Goats” story, at baseline and post-intervention for participants with aphasia.

	**PWA1**		**PWA2**		**PWA3**		**Mean (SD)**	
	**Pre-Tx**	**Post-Tx**	**Pre-Tx**	**Post-Tx**	**Pre-Tx**	**Post-Tx**	**Pre-Tx**	**Post-Tx**
***Elements***								
A1 setting	0	1	1	0	1	1	n/a	n/a
A2 protagonist	0	1	1	0	1	1	n/a	n/a
***Episode 1***								
A3 initiating event	0	1	0	0	0	0	n/a	n/a
A4 goal	1	0	1	0	0	0	n/a	n/a
A5 attempt	0	0	0	1	0	0	n/a	n/a
A6 outcome	1	1	1	1	0	0	n/a	n/a
A7 internal state	0	0	0	0	0	0	n/a	n/a
***Episode 2***								
A8 initiating event	0	1	1	1	0	0	n/a	n/a
A9 goal	1	0	1	1	0	0	n/a	n/a
A10 attempt	0	0	0	0	0	1	n/a	n/a
A11 outcome	0	1	0	1	0	0	n/a	n/a
A12 internal state	1	0	0	0	0	0	n/a	n/a
***Episode 3***								
A13 initiating event	0	1	1	1	0	0	n/a	n/a
A14 goal	0	0	0	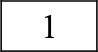	0	0	n/a	n/a
A15 attempt	1	1	1	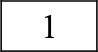	0	0	n/a	n/a
A16 outcome	1	1	1	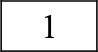	0	0	n/a	n/a
A17 internal state	0	1	0	0	0	0	n/a	n/a
***Total Elements /17***	**6**	**10**	**9**	**9**	**2**	**3**	5.67 (3.51)	7.33 (3.79)

PWA, person with aphasia. Bold values indicate the total scores. The boxes indicate the changes in number of elements.

The individual scores obtained from narrating the “Baby Goats” story yielded a variety of results in terms of the number of elements of the macrostructure produced by each participant. Specifically, no participant produced any complete episodes either at baseline or post-intervention. PWA3 produced only two macrostructure elements at baseline (the setting and the protagonist) and added a third element (attempt) post-intervention. The total scores from baseline were lower for all three PWA when compared to the macrostructure elements produced post-intervention. Interestingly, PWA2 managed to complete an episode (attempt-goal-outcome) during the narration of the 3rd episode of the “Baby Goats” story post-intervention.

To summarize, all three participants with aphasia failed to produce multiple plot elements during the narration of their personal stories and the “Baby Goats” story. Language samples were characterized mainly by single nouns (e.g., objects and subjects) and very few content verbs (e.g., subjects with no actions). Language samples post-intervention from each participant with aphasia narrating an episode of a personal story and an episode of the “Baby Goats” story are reported below. These language samples were selected based on several criteria to ensure relevance, representativeness, and richness of the data, while at the same time, maintaining rigor of the research process and ethical integrity.

PWA1 Personal Story

«Κυριακή φαΐ. Kοτόπουλο, πατάτες, κουπέπια. Κάθε μέραπίεννε (ημητ έρα) σταλαχανικά. Πολλά ωραία. Xαρούμενη». (Cypriot Greek)

“*Sunday lunch. Chicken, potatoes, salad. Every went to the vegetables (*Initiating Event). *Very nice. Happy*.”

PWA1 “Baby Goats”

«Τρία κατσίκιες και ένα κατσίδι μέστο νερό μέστο ποταμό. Τζιαιμια μάνα επίε μές το ποταμο. Κουντά το κατσίκι έξω. Μάνα κατσίκι». (Cypriot Greek)

“*Three goats and one goat in the water in the stream* (setting). *And one mother went into the stream* (initiating event). *Pushes the goat out* (outcome). *Mother, goat*.” (English translation)

PWA2 Personal Story

«Το πρωί της Κυριακής εγώ επίεννα εκκλησιά με την μάμμα μουτζιαι τον παπά μου. Μετά όταν ερκούμασταν σπίτι εφάμε, δαμέσα.. Τζιαι μετά η μάμμα μου έκαμε μέστο ψητό, κουπέπια, κολοκάσι, κιοφτέδες. Τζιαι μετά ετρώαμε» (Cypriot Greek)

“*Sunday morning I was going church. With my mother and my father* (setting/characters). Afterwards, *when we came home, we ate, in here* (goal). And *then my mother did in the roast, dolmades, taro root, meat balls*. *Then we were eating* (attempt).” (English translation)

PWA2 “Baby Goats”

«Αλλά μια πουτούντην πάστο δέντρο εθόρεν τους. Τζιαι εμούνταρεν να το πκιάει τζιαι μούνταρεν πουκάτο. Τζιαι ε έπκιασεν το δαμέ ε τζιαι σιγά σιγά έθκιωξεν τον η κατσίκα. Τζιαι τα μωρά εμείναν μαζί με την μάμμα τους». (Cypriot Greek)

“*She the thing on the tree was watching them* (initiating event). *And she hurtled to get it* (goal) *and she attacked underneath* (attempt). *And she catches it here, slowly slowly, the goat pushes him away; and the babies stay with their mother* (outcome)”. (English translation)

PWA3 Personal Story

“Που ήταν είκοσι χρονών έμαθαν χορούς και όλα αυτά τα παιδιά αυτά πήγαν με τους άλλους. Τα παιδιά μου εμείναν παρακολουθούσαν, οι ξένοι είχαν φύγει. Ίσως δεν τους άρεσε ή εμάθαν πολλά. Επεράσαμε πολλά ωραία. Χορούς ελληνικούς και κυπριακούς”. (Cypriot Greek)

“*When he was twenty years old (time/set) they learned dances and all these kids went away with the others* (initiating event). *My kids were watching* (goal), *the strangers left*. *Maybe they did not like it or they learned too much. We had a great time* (internal state). *Dances Greek and Cypriot*.” (English translation)

*PWA3 “Baby Goats”*

«Βλέπω δύο κατσίκες έξω από το νερό και μια κατσίκα μέσα στο νερό. Βλέπω τρία κατσικάκια και ένα λύκο να επιτίθετε». (Cypriot Greek)

PWA3 “Baby Goats”

«Βλέπω δύο κατσίκες έξω από το νερό και μια κατσίκα μέσαστο νερό. Βλέπω τρία κατσικάκια και ένα λύκο να επιτίθετε» (Cypriot Greek)

“*I see two goats out of the water and a goat in the water (setting). I see three baby goats and a wolf attacking (attempt)*.” (English translation)

Surprisingly, PWA3 who performed poorly during the telling of the “Baby Goats” story, had the highest performance, of the three participants with aphasia, on the narration of his personal story. Similarly, although PWA2 managed to complete an episode while narrating the third episode of the “Baby Goats” story, his performance improved considerably during the telling of his personal story. On the contrary, PWA1 showed no improvement during the narration of her personal story however, she made numerical gains after telling of the “Baby Goats” story post-intervention.

### Aphasia severity

The results revealed no change in scores on the Aphasia Severity Rating Scale (ASRS) for any of the participants with aphasia post-treatment. Despite presenting with mild-moderate language and communication difficulties prior to the treatment, no change in aphasic symptoms was observed after the intervention was completed.

### Functional communication

Functional communication was assessed with the Scenario Test-GR (Charalambous et al., 2022a). Results revealed numerical changes in functional communication for all three participants with aphasia after treatment. Specifically, PWA1 had a score of 36/54 at baseline and 42/54 at the post-intervention assessment. Functional communication for her was increased by 17%. Further, for PWA2 baseline score was 44/54, while the post-intervention score was 47/54, showing an 8% increase in functional communication. Participant PWA3 scored 35/54 at baseline and 44/54 post-intervention showing an increase of 22% in functional communication. Overall, across the participants with aphasia, the mean performance at baseline was 38.3 (sd = 4.93) and post-intervention 44.3 (sd = 2.52), indicating that functional communication had improved for all three participants after the intervention.

### Impact of aphasia on quality-of-life

The impact of aphasia on quality of life was measured by the Greek version of the Aphasia Impact Questionnaire-21 (Charalambous et al., 2022b). The results showed improvements in all three participants with aphasia given that the impact of aphasia was reduced post-intervention. Specifically, PWA1 had a baseline score of 40/84, which changed to 20/84 post-treatment, showing a 50% reduction of aphasia impact on QoL. PWA2 scored 31/84 at baseline, which was changed to 16/84 post-treatment, showing a reduction in the impact of aphasia on QoL by 48%. PWA3 scored 37/84 at baseline which changed to 10/84 post-treatment (72% reduction). The total mean score of the impact of aphasia on QoL for all three participants, at baseline and post-intervention was 36 (sd = 4.58), and 15.3 (sd = 5.03) respectively.

### Content validity of the group intervention

For the evaluation of the importance of the topics, the relevance of the materials, and the appropriateness of the intervention, a questionnaire on content validity was scored by all three participants with aphasia as described in the Method section. The scores for each participant in the different subcategories are presented in Table 8.

**Table 8:** Median scores of each participant in each category of the content.

	**Participants**	**Median**	**Percentiles**	
			**25th**	**75th**
Importance	PWA1	4.00	3.25	4.75
	PWA2	5.00	5.00	5.00
	PWA3	4.00	4.00	5.00
Comprehensiveness	PWA1	5.00	4.25	5.00
	PWA2	5.00	5.00	5.00
	PWA3	5.00	5.00	5.00
Relevance	PWA1	4.00	4.00	4.75
	PWA2	5.00	5.00	5.00
	PWA3	5.00	4.00	5.00
Appropriateness	PWA1	4.00	4.00	4.75
	PWA2	4.50	4.00	5.00
	PWA3	4.50	4.00	5.00

In total, content validity was given higher scores with an overall median score of 4. No between subjects differences were found for the scores of each category. Overall, the results confirm that participants with aphasia considered the content of the intervention relevant and appropriate.

## Discussion

This pilot study aimed to investigate whether the use of the codesign and coproduction approach in the development of a multilevel personal narrative intervention at the group level, increased production of macrostructure elements in trained and untrained narrative discourse contexts, improved aphasia severity and functional communication skills, and advanced quality of life of the participants with aphasia. Each research aim, put as a question, is addressed in the section below and the advantages of coproduction and codesign approaches to developing treatment activities are explored. The discussion section concludes with the clinical implications of the research.

*Research question 1: Was there an improvement in the personal narrative abilities of participants with aphasia after personal narrative intervention that was codesigned and coproduced with them?*

Production of coherent personal stories relies heavily on speakers' understanding of shared world knowledge and communicative resources in their culture (Olness and Ulatowska, 2011). This study took place in Cyprus, an island country in the Eastern Basin of the Mediterranean Sea, that is linguistically characterized by diglossia (Cypriot Greek dialect and Standard Modern Greek) and is bicultural (Greek Cypriot) (Fotiou and Grohmann, 2022). Overall, all three participants with aphasia showed a numerical improvement in their ability to narrate personal stories related to their childhood “House” story that was trained in treatment as evidenced by the production of additional story/plot elements and informational content, although no participant produced any complete episodes. PWA improved their ability to tell a complete and more informative story at the macrostructural level post-treatment, especially when narrating it to the group, although the exact measures that led to improvement varied. The results lend support to the findings from earlier studies investigating improvements in the macrostructure level of narratives after discourse treatment (Osiejuk, 1991; Carragher et al., 2015) and findings from studies that had implemented multilevel treatments to improve narrative discourse (Penn and Beecham, 1992; Whitworth, 2010; Whitworth et al., 2015).

*Research question 2*: *Did personal narrative intervention generalize to telling of a narrative story using a picture sequence that was untrained?*

The untrained narrative discourse task was the telling of the Baby Goats three-episode picture series story from the MAIN tool (Gagarina et al., 2019). The findings revealed numerical improvements in different components of story grammar (setting/protagonist/initiating event/attempt/outcome/internal state etc.) for each participant with aphasia. PWA1 showed the most numerical improvement compared to the others, as she was able to retrieve the highest number of elements in the story (setting and protagonist of episode 1, all three episode initiating events, outcome of episode 2, and internal state of episode 3) after treatment whereas PWA 2 produced an additional three elements post-intervention (attempt and outcome for episode 1 and goal for episode 3) and PWA3 only one more (attempt for episode 2).

*Research question 3: Were there gains in aphasia severity and functional communication for participants with aphasia post-intervention?*

This is the first study to our knowledge to incorporate a standardized measure of functional communication to measure any benefits of discourse treatment on communication in everyday life scenarios. Although aphasia severity remained the same for all three participants, numerical improvements in functional communication were observed as PWA successfully conveyed their responses to the scenarios using total communication strategies including movement, drawing, and gestures in an interactive context with the support of their communication partner.

*Research question 4: Were there a reduction in the impact of aphasia and gains in quality of life for participants with aphasia post-treatment?*

There was a numerical improvement in the reduction of the impact of aphasia on quality of life for all participants. Also, following the group intervention, PWA improved in the way they perceive the emotional consequences of aphasia in their everyday life compared to their baseline responses prior to the group intervention (Charalambous et al., 2022b). This is a worthy finding as it reinforces the benefits of codesigning and coproducing discourse treatment at the group level within an aphasia communication group where members share control and collectively decide on topics for practicing communication in a safe and friendly environment with peers. This is a clear message to clinicians to consider referring clients with aphasia on to aphasia communication groups also considered rope teams and social microcosmos (Lanyon et al., 2018).

*Research Question 5: What were the benefits of codesign and coproduction on treatment content and intervention outcomes?*

Participants reported that the codesign and coproduction methodology employed in the development of the treatment content prompted their active engagement during the 11-week study block. The participants with aphasia found the treatment content to be very relevant and worthwhile confirming that personal storytelling is crucial for social life networks and well-being (Strong and Shadden, 2020). Participants emphasized that improving their narrative skills was fundamental for everyday interactions with friends and family members. Moreover, verbal commands, explanations, and information provided to them for tasks and activities were clear and easily understood. Participants with aphasia confirmed that none of the materials or tasks used in the intervention were inappropriate or offensive. Furthermore, in the group setting, people with aphasia were pleased to receive additional information on aphasia, the types of services provided to them in group, and strategies around the general process of creating and narrating their own personal stories.

During the codesign and coproduction process, the impact on functional communication was observed at group discussions. The use of total communication strategies, allowed participants to utilize various communication techniques such as drawing, gestures, writing, visual prompts, etc. This motivated active participation in group conversations and tasks. The total communication approach also encouraged participants with aphasia to initiate discussions and share their perspectives on everyday topics like sports, hobbies, personal interests, and social media. Also, PWA reported that they were more confident when sharing their personal stories through both oral and non-verbal expression (use of pictographic support and gestures) and reported increased participation and involvement in everyday conversations with family members during mealtimes.

The communication partners (CPs) reported that following the structure of the PAOLI framework (Charalambous et al., 2023) assisted in organizing the induction meeting, informed on training, and preparing PWA on codesign and coproduction, and the overall protocol of the intervention. The CPs suggested various ways to create communication links with the PWA in the group. Also, the PAOLI helped CPs monitor how to actively involve PWA in conceptualizing the topics of the sessions, establish priorities and goals, and reach a consensus on how to approach the development of the personal narrative content around the “House” prop for everyone with aphasia. This helped with the overall approach of working with codesign methods. Also, the CPs were prompted to support PWA to self-monitor their performance while documenting the progress of the intervention.

### Clinical implementation

This pilot study is the first to use the codesign approach to develop a multilevel discourse treatment based on the LUNA protocol (Cruice et al., 2022), drawing content from the personal narratives of PWA. As such the findings should be considered preliminary. Personal narrative intervention aimed at improving the narrative skills of PWA resulting in an overall improvement in their quality of life with aphasia and increasing their participation in daily interactions. This confirms the relevant findings in the literature supporting that biographic-narrative intervention positively influences the identity of PWA and their overall quality of life (Corsten et al., 2015). Codesigning and coproducing the intervention helped to meaningfully engage PWA within the group sessions since the content of the tasks was related to their interests and preferences. This person-centered approach was also found to be effective in other discourse treatment studies (Osiejuk, 1991; Dietz et al., 2018; Cruice et al., 2022). Over the 9-week intervention, PWA were motivated to actively participate in the sessions as the focus was on creating and developing their personal stories. Improving the narrative skills of PWA encouraged them to participate more actively in shared activities within the group (Corsten et al., 2014). The codesign and coproduction methodology used in aphasia communication groups improved functional communication abilities that led to a positive impact on daily life. These approaches can be a valuable tool for rehabilitation specialists to cater to the needs of individuals with chronic aphasia in a group setting.

The findings of this pilot study are consistent with the phase three activities of the LUNA project as described by Cruice et al. (2022) relating person-centered and patient involvement approaches through codesign and coproduction and fostering active collaboration between communication partners (SLT clinicians) and people with aphasia. The present study was informed by the recent systematic review on discourse treatment studies in aphasia (Dipper et al., 2020) and the theoretical framework developed by Dipper et al. (2021) to select evidence-based practices for a group intervention protocol. Data was collected through spontaneous language samples, which were analyzed both quantitatively and qualitatively. Moreover, in conjunction with the coproduction protocol, we incorporated standardized tools tailored for Greek-speaking PWA. This integration allowed for the collection of quantitative data, enabling a comprehensive assessment of functional communication enhancement and the mitigation of the impact of aphasia on quality of life. The utilization of various techniques and approaches throughout the intervention period appears to mirror a dynamic aphasia group session in real-time, deviating from the conventional use of a singular intervention method. This divergence from a single-method approach aligns more closely with the pragmatic realities of speech-language therapy practice globally (Mason et al., 2020).

### Limitations

This pilot study has several limitations that are primarily methodological in nature. With only one baseline measurement taken pre-treatment it was not possible to proceed to statistical analysis of the results using, for example, WEighted STatistics (WEST) (Howard et al., 2015) to measure the significance of treatment effects. Also, the variation in performance between the two genres (tasks) indicates the different functions they serve. The difference in performance for PWA3 implies a disconnect between the two genres/tasks. It is worth noting that using a task such as telling a story with pictures to measure generalization is a limitation of this study. Telling a story with pictures to measure generalization is a limitation because it may not accurately reflect the broader communication abilities and real-world application of skills by PWA. Furthermore, due to logistical constraints, such as limited room availability and scheduling difficulties, we were unable to conduct assessments at multiple time points as recommended in discourse research. We acknowledge this limitation may have impacted the quality of our findings in relation to the reliability of the sampling. The study did not have a follow-up or maintenance phase making it impossible to determine how the intervention impacted on communication outside of treatment long-term. However, we recommend that PWA continue attending community-based aphasia communication groups to continue practicing discourse skills and to monitor post-therapy progress in a supportive environment (Charalambous and Kambanaros, 2021). Furthermore, role-playing common communication challenges and introducing communication aids and technological devices for communication will further promote independence (Charalambous and Kambanaros, 2021). Most importantly, fidelity was not directly assessed by the researchers. Fidelity in aphasia studies is crucial, irrespective of methodology, as it ensures that interventions are delivered as intended, allowing for accurate assessment of their effectiveness and reproducibility of results (Behn et al., 2023). Finally, the use of video recording solely for assessing verbal output overlooked the potential richness of non-verbal cues used during group and individual communication.

## Future directions

This pilot study is of a preliminary nature but leads to the need to find ways to identify, measure, code and treat discourse—as well as refining the coproduction techniques. Furthermore, there is a call for research to explore the timing and utilization of total communication strategies for conveying meaning, as observed in this study (Holland, 2021), given that the current assessment focused solely on verbal productions. Finally, clinical researchers may consider implementing this methodology in one-on-one sessions with individuals with aphasia to investigate whether individualized therapy prompts enhanced improvements in narrative skills and discourse. However, future studies with increased rigor are of paramount importance to inform principle-based interventions for people with aphasia.

Additionally, the study participants with aphasia expressed satisfaction with their enhanced understanding of aphasia and found the continuous support from communication partners in the group to be beneficial for overall performance. This underscores the importance of well-informed and aphasia-aware communication partners, suggesting that future studies should consider involving everyday caregivers in their intervention protocols when aiming for functional outcomes.

## Conclusion

The integration of codesign and coproduction methods and personal narrative content, present a promising strategy for enhancing the narrative skills of individuals grappling with chronic aphasia. In this pilot study, this combined approach demonstrated efficacy in advancing narrative proficiency, fostering improvement in functional communication, and positively impacting the overall quality of life for participants with aphasia. In essence, the consolidation of codesign principles, group collaboration, and evidence-based interventions in narrative discourse treatment development holds significant potential for augmenting functional communication and advancing quality of life throughout the chronic phase of aphasia. Prioritizing the enhancement of narrative skills emerges as a pivotal focus in the rehabilitation of individuals dealing with chronic aphasia. It is imperative to delve deeper into discourse treatment and for languages beyond English, to integrate group interventions into aphasia rehabilitation services globally, ensuring that they are tailored to meet the unique needs and preferences of individuals impacted by chronic aphasia.

## Data availability statement

The original contributions presented in the study are included in the article/Supplementary material, further inquiries can be directed to the corresponding author.

## Ethics statement

The studies involving humans were approved by Cyprus National Bioethics Committee (EEBK/ΕΠ/2017/37). The studies were conducted in accordance with the local legislation and institutional requirements. The participants provided their written informed consent to participate in this study. Written informed consent was obtained from the individual(s) for the publication of any potentially identifiable images or data included in this article.

## Author contributions

MC: Conceptualization, Data curation, Formal analysis, Investigation, Methodology, Project administration, Supervision, Visualization, Writing – original draft, Writing – review & editing. RS: Investigation, Project administration, Resources, Visualization, Writing – review & editing. ET: Data curation, Formal analysis, Methodology, Supervision, Writing – review & editing. MK: Data curation, Formal analysis, Methodology, Resources, Supervision, Visualization, Writing – review & editing.
