# Supplementary material for: The coproduction of a multilevel personal narrative intervention for people with aphasia in a community communication support group—A pilot study
Source: Front Stroke. 2024 Jul 17;3:1393676. doi: 10.3389/fstro.2024.1393676 (PMC12802638; doi:10.3389/fstro.2024.1393676)
Supplement: Supplementary file 2 [file Data_Sheet_2.docx]

**Supplementary Material**

Appendix 2

### Table 10. A description of the 9-week treatment protocol in relation to the role of the moderator and the CPs, what was expected of the participants with aphasia, the codesign methods used, and the communication strategies and therapeutic approaches.

| **Week** **#** | **Intervention level** | **Moderator** | **Persons with aphasia (PWA)** | **Communication Partners (CPs)** | **Codesign methods** | **Communication Strategy** | **Facilitation Techniques & Therapeutic approaches** |
| --- | --- | --- | --- | --- | --- | --- | --- |
| 2 | Word level treatment   - Introduction on Codesign Methods and Narrative Skills enhancement in aphasia - Discussions on everyday communication difficulties while living with chronic aphasia | - Gave aphasia friendly information to PWA about the project, commitments, and their role during the codesign - Gained signed informed consent for participation, audio and video recording - Presented information on the codesign methodology through an aphasia-friendly PowerPoint - Recorded and transcribed the personal stories | - Introduced themselves - Provided information about their stroke story and shared personal experiences of the everyday communication challenges of chronic aphasia | - Supported PWA on auditory comprehension on the meaning of the task/concept - Refocused PWA on the topic/task | - Co- decision and extraction of single words that will be used during the tasks and activities (again based on personal stories) - Co-decision on communication strategies and facilitation techniques to be used e.g. use of visual support, gestures etc. - Shortlisted 3 types of visual prompts to be used: linear black and white infographics, real photos, and the Mulberry symbols. | - Visual prompts - Writing - Gestures - Pointing | Phonological and semantic cueing |
| 3 | Word level treatment | - Gave a summary of the previous session/ presented the minutes in an Aphasia-friendly format on PowerPoint. - Explained the therapeutic approaches used at the word level e.g. PACE - Kept notes - Recorded the session - Coordinated the flow of the session - Signaled breaks - Recorded all task responses | - Constructed their “House” with papers and scissors. - Named family members, friends, nouns, and verbs. - Selected and used relevant Mulberry symbols. | - Recorded the selected vocabulary for each PWA and created visual prompts - Prepared pictures of nouns and verbs related to each "House” - Supported PWA when needed by rephrasing the command or refocusing PWA on the task - Helped PWA to paste pictures in the “House” | - Personalized vocabulary based on each personal story. These included nouns and verbs related to the "House” (e.g., dog, Pelendri, Marathovouno, family, grandma, cousins, hunting, soccer, friends) - Selection of Mulberry Symbols | - Visual prompts - Writing - Gestures - Pointing | - Phonological and semantic cueing - PACE |
| 4 | Word level treatment | - Gave a summary of the previous session/ presented the minutes in an Aphasia-friendly format on PowerPoint. - Explained the PACE technique - Keeping notes - Recording the session - Coordinating the flow of the session - Indicating breaks - Recording responses | - PWA named verbs - PWA performed actions to express verbs using PACE | Presented verbs in pictures | Personalized vocabulary based on each personal story. These included the basic verbs of each personal story of the “House” e.g., eat, drink, play, work, study | - Visual prompts - Writing - Gestures - Pointing | - Phonological and semantic cueing - PACE |
| 5 | Sentence level treatment | - Gave a summary of the previous session/ presented the minutes in an Aphasia-friendly format on PowerPoint. - Explained the NARNIA diagram, PACE and M-RET - Kept notes - Recorded the session - Coordinated the flow of the session - Indicated breaks - Recorded all responses to the tasks | Repeated sentences with SVO structure | - Presented out loud an SVO sentence related to personal stories e.g. Mother prepared meal - The NARNIA diagram was provided to each PWA in a printed format and was used when repeated their sentences related to their personal stories | - Co-selected and coproduced sentences based on each personal story. These were informed by the nouns and verbs produced by PWA during the narration of each personal story of the “House” - Addition of pronouns, articles e.g. “She went to the supermarket” | - Visual prompts - Writing - Gestures - Pointing | - PACE - NARNIA - M-RET |
| 6 | Sentence level treatment | - Gave a summary of the previous session/ presented the minutes in an Aphasia-friendly format on PowerPoint. - Explained NARNIA and PACE - Kept notes - Recorded the session - Coordinated the flow of the session - Indicated breaks - Recorded all responses to the tasks | - Described picture using a single sentence | - Presented complex picture related to personal stories - Use of the NARNIA diagram approach for sentence generation as mentioned above   This included the gradual addition of elements in the sentences with Wh- questions: Who, Where, When, What, Why, How. | - Co-assembled Mulberry symbols to create picture setting - Coproduced sentences based on each personal story of the “House”. These included the verbs and nouns from each personal story e.g. “My mother cooked roast” | - Visual prompts - Writing - Gestures - Pointing | - Semantic cueing - PACE - NARNIA |
| 7 | Sentence level treatment | - Gave a summary of the previous session/ presented the minutes in an Aphasia-friendly format on PowerPoint. - Explained ORLA and M-RET - Kept notes - Recorded the session - Coordinated the flow of the session - Indicated breaks - Recorded all responses to the tasks | - Read sentences with SVO structure using ORLA - Created SVO sentences to respond | - Wrote sentences with SVO structure - Prompted PWA to create SVO sentences with visual cues (mulberry symbols from the House). | All sentences where codesigned and coproduced based on personal stories as mentioned above | - Visual prompts - Writing - Gestures - Pointing | - ORLA - M-RET |
| 8 | Discourse level treatment  (Macrostructure) | - Gave a summary of the previous session/ presented the minutes in an Aphasia-friendly format on PowerPoint. - Explained NARNIA, PACE, ORLA, and M-RET - Kept notes - Recorded the session - Coordinated the flow of the session - Indicated breaks - Recorded all responses to the tasks | - Described pictures based on the constructed paper “House” - Narrated sequences of everyday events via photo cards | - Initiated open discussions based on personal stories e.g. “Tell me an event that happened in your House” - Prompt PWA to put cards in the write order (3-step sequences) and narrate story | - All open discussion topics were selected by PWA e.g. lunch time, going to the church, reading homework - All story cards were co-selected with PWA e.g. watching football, having a haircut | - Visual prompts - Gestures - Pointing | - NARNIA - PACE ORLA - M-RET |
| 9 | Discourse level treatment  (Macrostructure) | - Gave a summary of the previous session/ presented the minutes in an Aphasia-friendly format on PowerPoint. - Explained NARNIA, ORLA, and M-RET, - Kept notes - Recorded the session - Coordinated the flow of the session - Indicated breaks - Recorded all responses to the tasks | - Making hypothetical discussions using a script and role-playing - Watched a video of self-narrating the personal story and reflected on narrative skills, naming errors etc. | - Asked PWA to respond to a hypothetical scenario from the perspective of another group member. - Prompted PWA to self-reflect in various ways e.g. using gestures and pointing pictures | Hypothetical scenarios were coproduced with PWA | - Visual prompts - Gestures - Pointing | - SWIM - Self-Monitoring |
| 10 | Discourse level treatment  (Macrostructure) | - Gave a summary of the previous session/ presented the minutes in an Aphasia-friendly format on PowerPoint. - Explained NARNIA, Script Training and SWIM - Transcribed and provided the personal stories’ scripts to the communication partners - Kept notes - Recorded the session - Coordinated the flow of the session - Indicated breaks - Recorded all responses to the tasks | - Retold personal stories - PWA mimicked specific scenarios they chose related to their daily lives in relation to their “House” e.g. going to the supermarket, church, hunting etc. | - Each communication partner told the personal story of each person with aphasia based on their “House.” - Provided visual representation of daily scenarios. - Asked PWA to respond to a scenario mentioned by another PWA from the perspective of another group member - Assisted PWA to practice the role play - Supported PWA to engage in role play | - Stories were coproduced in previous sessions - Scenarios were co-developed with PWA in previous sessions - Role play was co-devised by PWA and their communication partners | - Visual prompts - Writing - Gestures - Pointing | - Script Training - NARNIA - SWIM |

**Note: M-Ret= Modified Response Elaboration Training, PACE= Promoting Aphasics' Communicative Effectiveness, ORLA= Oral Reading for Language in Aphasia, NARNIA= Novel Approach to Real Life Communication, SWIM= "Someone Who Is Not Me", CPs= Communication Partners**
