# Supplementary material for: The coproduction of a multilevel personal narrative intervention for people with aphasia in a community communication support group—A pilot study
Source: Front Stroke. 2024 Jul 17;3:1393676. doi: 10.3389/fstro.2024.1393676 (PMC12802638; doi:10.3389/fstro.2024.1393676)
Supplement: Supplementary file 3 [file Data_Sheet_3.pdf]

Supplementary Material

Appendix 3

Figure 3 A screenshot of the questionnaire providing an example of the scale and the presentation of the questions used in the content validity study.

14.Τα θέματα που συζητούσα ήταν κατάλληλα για μένα. *

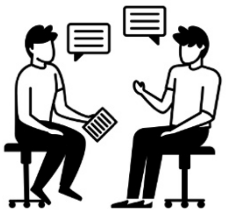

	1 ☹️ Διαφωνώ ...	2 🙁 Διαφωνώ	3 😐 Ουδέτερο	4 🙂 Συμφωνώ	5 😊 Συμφωνώ...
Σημειώνω	☐	☐	☐	☐	☐
